# Tephritid-microbial interactions to enhance fruit fly performance in sterile insect technique programs

**DOI:** 10.1186/s12866-019-1650-0

**Published:** 2019-12-24

**Authors:** Ania T. Deutscher, Toni A. Chapman, Lucas A. Shuttleworth, Markus Riegler, Olivia L. Reynolds

**Affiliations:** 1 Biosecurity and Food Safety, NSW Department of Primary Industries, Elizabeth Macarthur Agricultural Institute, Private Bag 4008, Narellan, 2567 New South Wales Australia; 2 Graham Centre for Agricultural Innovation (an alliance between NSW Department of Primary Industries and Charles Sturt University), Elizabeth Macarthur Agricultural Institute, Private Bag 4008, Narellan, 2567 New South Wales Australia; 3 Current address: NIAB EMR, Department of Pest and Pathogen Ecology, East Malling, Kent, ME19 6BJ UK; 4 Hawkesbury Institute for the Environment, Western Sydney University, Locked Bag 1797, Penrith, 2751 New South Wales Australia; 5 Current address: cesar Pty Ltd, 293 Royal Parade, Parkville, Victoria 3052 Australia

**Keywords:** Tephritidae, SIT, Gut microbiota, Gut microbiome, Host-microbe interaction, Insect microbial symbiosis, Microbial symbiont, Probiotics, Mass-rearing

## Abstract

**Background:**

The Sterile Insect Technique (SIT) is being applied for the management of economically important pest fruit flies (Diptera: Tephritidae) in a number of countries worldwide. The success and cost effectiveness of SIT depends upon the ability of mass-reared sterilized male insects to successfully copulate with conspecific wild fertile females when released in the field.

**Methods:**

We conducted a critical analysis of the literature about the tephritid gut microbiome including the advancement of methods for the identification and characterization of microbiota, particularly next generation sequencing, the impacts of irradiation (to induce sterility of flies) and fruit fly rearing, and the use of probiotics to manipulate the fruit fly gut microbiota.

**Results:**

Domestication, mass-rearing, irradiation and handling, as required in SIT, may change the structure of the fruit flies’ gut microbial community compared to that of wild flies under field conditions. Gut microbiota of tephritids are important in their hosts’ development, performance and physiology. Knowledge of how mass-rearing and associated changes of the microbial community impact the functional role of the bacteria and host biology is limited. Probiotics offer potential to encourage a gut microbial community that limits pathogens, and improves the quality of fruit flies.

**Conclusions:**

Advances in technologies used to identify and characterize the gut microbiota will continue to expand our understanding of tephritid gut microbial diversity and community composition. Knowledge about the functions of gut microbes will increase through the use of gnotobiotic models, genome sequencing, metagenomics, metatranscriptomics, metabolomics and metaproteomics. The use of probiotics, or manipulation of the gut microbiota, offers significant opportunities to enhance the production of high quality, performing fruit flies in operational SIT programs.

## Background

Worldwide, fruit flies (Tephritidae) annually cause substantial damage to horticultural crops, and limit domestic and international trade. Some of the most economically important tephritids include the Mediterranean fruit fly (*Ceratitis capitata*), oriental fruit fly (*Bactrocera dorsalis*) and Queensland fruit fly (*Bactrocera tryoni*). Sterile Insect Technique (SIT) is currently employed in a number of countries to prevent, suppress, contain or eradicate targeted pest species, including tephritid fruit flies [1]. SIT is most successful in an area wide - integrated pest management (AW-IPM) scenario, or geographic isolation [2, 3], and when used in conjunction with other management techniques [4, 5]. The success of SIT depends on irradiated sterile male insects effectively locating, attracting and successfully copulating with wild females [6]. This approach has several advantages including that it is sustainable, has low impact on the environment, does not involve insecticides, and is target-specific.

Fruit fly domestication, irradiation, mass-rearing and handling reduce the fitness, performance and longevity of flies used in SIT programs, thereby reducing the effectiveness of SIT and its cost-benefit ratio [7–9]. Behavioural and physiological changes of mass-reared sterile males, such as changes in mating time and duration, ability to join leks, courtship rituals, pheromone production and attractiveness compared to wild fertile males, dramatically affect copulatory success with wild females [8, 10]. Post-mating factors, such as ejaculate transfer and the inability to prevent re-mating, also influence copulatory success [11]. To overcome the typically low copulatory success of sterile males, a larger number of sterile flies are released, relative to the number of wild flies in the field [10, 12], resulting in high mass-rearing costs. Understanding the biology, ecology and behaviour of fruit flies and the effects of domestication, mass-rearing, handling and sterilization of target pest species allows optimization, and improves the cost, efficiency and effectiveness of SIT.

The gut microbiome greatly influences insect health and homeostasis [13, 14]. The symbiotic association of tephritids with bacteria has been recognized for over a century [15], but our appreciation of the importance and complexity of tephritid-microbial symbiont interactions has increased considerably over the last 35 years. Studies removing, or significantly reducing tephritid gut microbiota through antibiotics indicate that microbiota can positively influence various aspects of tephritid biology, such as nitrogen metabolism, longevity, reproduction, fecundity and overcoming phenolic fruit compounds [16–20]. For example, in contrast to antibiotic-fed (asymbiotic) adult olive fly (*Bactrocera oleae*), untreated flies were able to utilize inaccessible sources of nitrogen, and bacteria assisted in the provision of missing essential nutrients to the host [20]. Offspring of antibiotic-fed field caught *B. oleae* females failed to complete larval development in unripe olives unlike larvae of untreated females; however, both were able to complete development in ripe olives. Therefore, it was postulated that symbiotic bacteria help overcome the phenolic compounds in unripe olives [19]. A less intuitive example was found for *C. capitata*. Adults of this species treated with antibiotics and fed a sugar-only adult diet had significantly increased longevity compared with non-antibiotic-treated flies on the same diet; however, the same effects were not seen when the flies were fed a full adult diet (sugar and yeast hydrolysate) [17]. The authors suggested that the antibiotics may be aiding the immune system against non-beneficial gut microbiota of nutritionally stressed flies [17]. Further, an important trait that maintains gut microbiota in flies is their transmission across generations. Female tephritids coat the egg surface with bacteria prior to, or during oviposition, which aids larval development [21–25]. Fitt and O’Brien [26] found surface sterilization of eggs significantly reduced larval weight (3 mg) at 10 days, while larvae from eggs that were not surface sterilized grew normally, weighing about 15 mg. Studies adding symbiotic bacteria to artificial larval diets significantly improved the development and fitness of domesticated fruit flies [26–28]. Thus, the tephritid-microbe symbiotic relationships are very intricate and of significant ecological and evolutionary importance. Increasing our knowledge of these relationships may identify ways to enhance performance of insects that are mass- reared for SIT programs.

Our review focuses exclusively on tephritid gut symbionts, excluding intracellular endosymbionts, such as *Wolbachia,* which may also be detected in insect gut microbiome studies [29]; however, a previous study suggested that fewer tephritid species than expected harbour *Wolbachia* [30]. While previous review papers have mostly focused on specific tephritid species [31, 32], or progress in understanding the function of tephritid gut microbiota [33, 34], our review examines recent progress on methods and identification of tephritid microbial symbionts, the impact of the domestication process and irradiation on tephritid-microbial symbiont associations and the use of probiotics to manipulate the fruit fly gut microbiota and consequently gut health.

### Tephritid gut microbiota

#### Influence of methodology and sampling design

Current characterization techniques of tephritid gut microbial communities have advantages and limitations. Culture-dependent approaches select for microbes capable of growing under culturing conditions, with a large number of bacterial diversity still unculturable. Molecular methods enable the detection of both culturable and unculturable bacteria, rare bacteria and other difficult to culture microorganisms. Molecular approaches used in tephritid gut microbiome studies have targeted the 16S rRNA gene, and are rapidly expanding our knowledge of tephritid gut bacteria. Indeed, sequencing of 16S rRNA gene amplicons from DNA extracted from oesophageal bulbs of *B. oleae*, enabled the identification of the unculturable symbiont “*Candidatus* Erwinia dacicola” [35] that assists larvae developing in unripe olives to overcome the plant’s chemical defense mechanism [19].

Tephritid 16S rRNA gene NGS microbiome studies provide a more comprehensive view of fruit fly gut bacterial communities than earlier methods; however, in general each microbiome study employing NGS needs to be interpreted with some caution [36]. For example, 16S rRNA gene amplicon NGS of wild and laboratory-reared tephritids (larvae and adults) have found up to 24 operational taxonomic units (OTUs) at 97% sequence similarity [19, 20, 22, 37] (Table 1). These studies indicate that the tephritid microbiome is low in diversity, similar to that of *Drosophila* [43, 44]. However, two studies have reported much higher numbers of OTUs (97% similarity) when studying the gut microbiome of tephritid fruit fly samples; up to 322 OTUs for *Bactrocera minax* [39] and up to 81 OTUs for *B. dorsalis* [38] within a life stage time point. These large numbers of OTUs may, for example, be due to the number of samples pooled (50 samples were pooled in Andongma et al. [38]), quality trimming and/or clustering algorithms. Differences also appear to arise based on whether OTUs with low read numbers were discarded. For example Ben-Yosef et al. [19] removed OTUs with less than 10 sequences. No such restrictions were put on the total number of OTUs from various life stages reported in Andongma et al. [38]; however, employing the same criteria would result in a reduction in the total number of OTUs from combined life stages studied from 172 to 42. It is unclear whether OTUs with low read numbers were also removed from Wang et al. [39] and whether possible erroneous OTUs due to sequencing artefacts were removed from the pyrosequencing data; such erroneous OTUs were removed in Morrow et al. [37]. Nonetheless, discounting low prevalence organisms may also be risky, as microbes at low titers may be overlooked [45]. Furthermore, the percentage of sequence similarity used to define OTUs can alter the taxonomic microbiome profile. For example at > 97% similarity, larvae and adult *C. capitata* shared a dominant OTU, but this was not true when OTUs were called at > 98% similarity [22]. In regard to taxonomic resolution, the region of the 16S rRNA gene sequenced and the length of sequences obtained using NGS technologies is another factor that can confound analyses [46–50]. No two tephritid NGS microbiome studies have followed the same sequencing and analytical approaches (Table 1), which can complicate comparisons between studies, thus clear archiving of sequence data and reporting of downstream processing of the data (e.g. scripts) are critical.

**Table 1:** Summary of methodologies employed and results (reads and OTUs) of tephritid NGS microbiome studies

Study	Fly Species	Wild (i.e. field) or Domesticated (i.e. laboratory)	Life Stage	Tissue	Sequencing Method	Primers	Pipeline	Number of Samples	Number of Reads after Quality Control	OTUs (97% similarity)
Morrow et al. [37]	*B. tryoni*	laboratory	adult	whole flies	454 Pyrosequencing	341f 806r	QIIME	3 pools of 8	3019, 5994, 5991	10,7,14
	*B. tryoni*	field (citrus)	adult	whole flies				1 pool of 8	6133	16
	*B. neohumeralis*	laboratory	adult	whole flies				3 pools of 8	4761, 6741, 6344	6, 18, 14
	*B. jarvisi*	laboratory	adult	whole flies				1 pool of 8	13,200	7
	*B. cacuminata*	laboratory	adult	whole flies				1 pool of 8	8202	4
	*B. cacuminata*	field (wild tobacco)	adult	whole flies				1 pool of 8	7357	8
	*C. capitata*	laboratory	adult	whole flies				1 pool of 8	7134	1
	*D. pornia*	field (citrus)	adult	whole flies				1 pool of 8	8022	17
Aharon et al. [22]	*C. capitata*	field (apricot)	adult	midgut	454 Pyrosequencing	926f 1392r	Mothur	5 individual adults	5000-12000	5-23
		field (apricot)	larval	midgut				15 pooled as 3 samples	1700-3200	7-13
Andongma et al. [38]	*B. dorsalis*	field (healthy fruit)	eggs	whole egg	454 Pyrosequencing	27f 533r	Mothur	approx. 50 insects pooled	Total reads 46332 (lowest number of reads per sample was 5967)	76
		field (healthy fruit)	first instar	whole larva				as above		77
		field (fallen fruit)	third instar	whole gut (proventriculus to rectum)				as above		81
		field (3^rd^ instar larvae collected from fallen fruit allowed to pupate in a laboratory)	pupae	whole pupa (without puparium)				as above		60
		field (ME traps) ^a^	adult (F)	whole gut (proventriculus to rectum)				as above		59
		field (protein traps)	adult (M)	whole gut (proventriculus to rectum)				as above		54
Ben-Yosef et al. [19]	*B. oleae*	field (unripe and ripe 'Souri' olives)	third instar	gastric caeca at the proximal section of the midgut	Illumina MiSeq	515f 806r	Mothur	5 individuals per unripe and ripe olives	73868 ± 8677 reads per sample	5.4 ± 3.4^c^
		field (ovipositing in unripe and ripe 'Souri' olives)	adult (F)	midgut and esophageal bulb				4 individuals (ripe olives) 5 individuals (unripe olives)	as above^b^	as above^b^
		mass-reared (ovipositing)	adult (F)	midgut and esophageal bulb				6 individuals	as above	not reported
Ben-Yosef et al. [20]	*B. oleae*	field (green “Manzanillo” olives)	third instar	gastric caeca at the anterior section of the midgut	454 Pyrosequencing	926f 1392r	Mothur	5 individuals	8351 - 15,098 sequences per sample	1-2 per sample
		field (soil under olive trees)	pupae	midgut and esophageal bulb				5 individuals	6596-18,335 sequences per sample	1-3 per sample
		field collected pupae eclosed in cage	adult (F)	midgut and esophageal bulb				5 individuals	8344 - 12,599 sequences per sample	1-2 per sample
Wang et al. [39]	*B. minax*	field (citrus)	adult (F)	intestine	454 Pyrosequencing	343f 798r	DOTUR	3 pools of 15	7857	319
		field (citrus)	adult (F)	ovaries				3 pools of 15	8124	415
		field (citrus)	adult (M)	intestine				3 pools of 15	7353	322
		field (citrus)	adult (M)	testes				3 pools of 15	8957	389
Yong et al. [40]	*B. carambolae*	field (ME traps)^a^	adult (M)	whole fly	Illumina MiSeq	341f	MEGAN5	4 individuals	1,561,203 – 2,077,403	44-75 genera
	*B. dorsalis*	field (ME traps)^a^	adult (M)	whole fly		518r		2 individuals	1,584,084 – 1,607,064	55-75 genera
Ventura et al. [41]	*A. ludens*	field (bitter orange)	third instar	whole gut	454 Pyrosequencing	8f 556r	QIIME	30 insects pooled	110,073 reads	72
		field (CeraTrap®)	adult	whole gut				as above		121
	*A. obliqua*	field (mango)	third instar	whole gut				as above		38
		field (CeraTrap®)	adult	whole gut				as above		100
	*A. serpentina*	field (mamey sapote)	third instar	whole gut				as above		82
		field (CeraTrap®)	adult	whole gut				as above		121
	*A. striata*	field (guava)	third instar	whole gut				as above		75
		field (CeraTrap®)	adult	whole gut				as above		57
Malacrinò et al. [42]	*C. capitata*	field (orange)	first instar	whole larvae	Illumina MiSeq	515f 806r	QIIME	15 individuals	not specified	a total of 3,169
		field (orange)	third instar	whole larvae				as above		
		field larvae pupate in laboratory (orange)	pupae	whole pupae				as above		
		from field collected larvae (orange)	adults	whole flies				as above		
		field (fig)	third instar	whole larvae				as above	not specified	a total of 1,118
		field (prickly pear)	third instar	whole larvae				as above		
		field (peach)	third instar	whole larvae				as above		
		field (cherimoya)	third instar	whole larvae				as above		
		field (orange fruits)	third instar	whole larvae				as above		

^a^*ME* methyl eugenol

^b^average calculated from all field samples, i.e. both larvae and adults

^c^>10 reads per OTU and OTU clustered at 98% similarity

Very few common or ‘core’ bacteria at the genus or species level have been identified in tephritid gut microbiome studies. “*Ca.* E. dacicola” (Enterobacteriaceae) and *Acetobacter tropicalis* (Acetobacteraceae) have been identified as prevalent and possible ‘core’ bacteria in *B. oleae*; however, recent NGS studies of gut microbiota in *B. oleae* have failed to detect *A. tropicalis* in the samples analyzed [19, 20], possibly due to sampling of different host populations. The identity of core bacteria has probably also been overlooked as often tephritid gut microbiome studies have only analyzed pooled or small numbers (fewer than seven) individual samples, such as Andongma et al. [38], Morrow et al. [37], Ventura et al. [41], Wang et al. [39], Ben-Yosef et al. [19], Ben-Yosef et al. [20] and Yong et al. [40]. Furthermore, analysis of single pools of samples does not provide any information about diversity within a population. An exception is the *C. capitata* microbiome study by Malacrinò et al. [42], where 15 or more individuals per life stage were analyzed; however, whether any core bacteria were identified was not discussed. Increased studies on the bacterial diversity within and between populations can provide insight into the environmental influences on tephritids.

#### Tephritid bacterial communities

To date, the majority of studies investigating tephritid gut bacterial communities have focused on adults. Bacteria of tephritid larvae and changes across tephritid ontogeny have been characterized in few studies [19, 22, 38, 42, 51]. Bacterial complexity is lower at larval and pupal stages, but increases during the adult stage [22, 51], and likely reflects that the larval stage is naturally confined to a single fruit. There does not appear to be major differences in the bacterial classes or families present in the larval and the adult stage [22, 38]; however, relative abundances of bacterial families may shift with development [38]. This suggests that adult flies acquire microbiota in the larval and early teneral stages, although changes between life stages may be more pronounced when looking at the bacterial genus and species levels. Unfortunately, in many studies the short NGS reads combined with the polyphyly of Enterobacteriaceae has limited the resolution of taxa to these levels when analyzing them across developmental stages [22]. Current laboratory-based evidence suggests that once acquired, tephritid gut microbiota may remain relatively stable throughout adult fly development. The same bacterial species were still recoverable from a *B. tryoni* population 13 days after the bacteria were fed to the flies [52]. Furthermore, fluorescently labelled *Enterobacter agglomerans* and *Klebsiella pneumoniae* fed to adult *C. capitata* remained detectable in three successive generations of adult flies [21].

The majority of bacteria associated with tephritids belong to the phyla Proteobacteria or Firmicutes, with the most abundant and prevalent from only a few families. Studies of culturable and non-culturable bacteria of field collected tephritids revealed that Enterobacteriaceae are dominant in the vast majority of tephritids, including *C. capitata* [22, 37, 51, 53–57], Anastrepha spp. [41, 58], *Bactrocera* spp*.* [23, 26, 35, 37, 39, 40, 52, 59–69], *Rhagoletis* spp. [70, 71], and others. Further, Enterobacteriaceae dominate the bacteria vertically transferred from adult tephritid females to larvae, via coating of the egg surface with bacteria prior to, or during oviposition [21–25]. Morphological characteristics and behaviour of fruit flies, which contribute to both vertical and horizontal transmission of Enterobacteriaceae, suggests that these bacteria play an important role in fruit fly development and physiology.

Known functions of tephritid gut bacteria within the Enterobacteriaceae family include diazotrophy and pectinolysis [20, 22, 51, 53, 72], and the break-down of chemical host plant defenses [19] and insecticides [73]. However, there does not appear to be a common species or genus within the Enterobacteriaceae family that is consistently found in the studied tephritids or even within a fruit fly species, with the exception of “*Ca.* E. dacicola”, which is prevalent in all wild *B. oleae*. This phenotypic plasticity of gut microbiota could indicate that a number of bacteria can perform similar roles, which are conserved at higher taxonomic levels, and are interchangeable, thereby allowing tephritids to adapt to diverse diets, and changing bacterial communities.

Other commonly reported Proteobacteria belong to the families Pseudomonaceae and Acetobacteraceae. Pseudomonaceae are present in a number of tephritid species. For example, *Pseudomonas* constitutes a minor but stable community within the gut of *C. capitata*; however, at high densities *Pseudomonas aeruginosa* significantly reduces *C. capitata* longevity [54]. Therefore, the role of *Pseudomonas* spp. in tephritids remains unclear. The acetic acid bacteria *A. tropicalis* was reported as a major symbiont in *B. oleae* via a specific end-point PCR, but, as mentioned earlier, has not been detected in *B. oleae* 16S rRNA gene amplicon NGS studies [19, 20]. Acetobacteraceae have also been reported at low levels in other adult tephritids, but were highly abundant in a single pool of adult female *Dirioxa pornia* [36], a tephritid species with a particular ecological niche, infesting and developing in damaged and fermenting fallen fruit. Apart from research into *A. tropicalis* in *B. oleae*, very little attention has been given to the presence of acetic acid bacteria in tephritids, even though such bacteria are frequently reported as symbionts of insects that have a sugar-based diet within the orders Diptera (including *Drosophila* fruit fly species), Hymenoptera and Hemiptera [74].

Firmicutes constitute part of the microbiota of most adult *Bactrocera* spp. studied to date. Bacteria of the order Bacillales have been reported in *Bactrocera zonata* [68], and in *B. oleae* [75], and bacteria of the order Lactobacillales have been identified in *B. tryoni* [37, 64, 65], *B. minax* [39], *Bactrocera cacuminata* [64], *Bactrocera neohumeralis* [37], *B. oleae* [75] and *B. dorsalis* [38, 62]. Firmicutes have not frequently been reported for *C. capitata*, although *Leuconostoc* were recently detected in the *C. capitata* NGS microbiome study by Malacrinò et al. [42]. Lactobacillales were more common in laboratory-reared than field collected *Bactrocera* spp. flies [37]. Most Firmicutes stain Gram positive, and Gram positive bacteria are known to possess a number of mechanisms that increases their survival in acidic environments [76]. This could increase their tolerance of the low pH of larval diets, and, therefore, be carried on to the adult stage. In addition, some lactic acid bacteria are known to produce antimicrobial peptides [77], which may influence the presence of other bacteria in the diet and gut. The function of lactic acid bacteria in tephritids remains unknown.

### Fruit fly rearing in an artificial environment impacts on gut microbiota

Fruit flies reared in an artificial environment are not exposed to bacteria typically found in their natural habitat, including microbes that could confer fitness benefits. Artificial tephritid adult diets used for mass-rearing (colony maintenance, not pre-release diets) normally only comprise sugar and yeast hydrolysate; while larval diets typically comprise a bulking agent, yeast, carbohydrates (in the form of sugar or other carbohydrates either added, or within the bulking agent) and antimicrobial agents, such as antifungal and antibacterial agents [78]. While the antimicrobial agents and pH of the larval diet reduce the possibility of contamination with detrimental microorganisms, they may also reduce the opportunities for horizontal transmission of beneficial microbes. Similarly, egg collection methods that rely on water as a transfer medium, and handling methods (e.g. bubbling at temperatures to induce female mortality; required for temperature sensitive lethal strains to produce male only flies under SIT programs), may allow the wider spread of pathogenic bacteria across cultures, and also reduce the vertical transmission of beneficial microorganisms from the adult through to the larval stage.

Consequently, tephritid rearing can change gut microbial communities by reducing bacterial diversity relative to field-collected specimens [19, 24, 37], altering the relative abundance of particular microbes [56] and promoting the acquisition of bacterial species not commonly found in field flies [19, 37]. Mass-reared larvae also have a lower bacterial load than their wild counterparts; larvae from mass-reared olive flies developing in olives have a comparable bacterial load to larvae from field-collected olive flies treated with antibiotics [19]. In addition, olive flies fed an artificial diet have been shown to specifically lack the bacterial symbiont “*Ca.* E. dacicola”, found in wild flies [59], while artificially reared olive flies fed on olives retain the symbiont [19]. This bacterium allows larvae to develop in unripe olives by counteracting the effects of the phenolic glycoside oleuropein [19]. Although this function is no longer necessary for olive flies not reared on olives, “*Ca*. E. dacicola” can also accelerate larval development, perhaps through the provision of nitrogen [19]. In contrast, mass-reared adult female olive fly guts were dominated almost exclusively by *Providencia* spp. [19]. Similarly, while *Pseudomonas* spp. occur at only low levels in field collected *C. capitata* (~ 0.005% of total gut bacteria) [54], they can constitute more than 15% of the total gut bacterial population of mass-reared adult Vienna 8 *C. capitata* [56]. The relative abundance of Enterobacteriaceae in laboratory-reared adult *B. tryoni* colonies was reduced compared to field collected *B. tryoni*; however, only three pools of laboratory-reared *B. tryoni* from different populations were compared to just one pool of field collected *B. tryoni*, and only females were analyzed [37]. Laboratory rearing also influences the abundance of lactic acid bacteria, such as *Lactococcus*, *Vagococcus and Enterococcus* in some *Bactrocera* laboratory-adapted flies, which do not tend to be present in high densities in wild flies [37].

The gut microbiota of fruit flies also become very similar and ‘streamlined’ when maintained on the same diet within a location. Adult *B. tryoni*, sourced from different locations maintained on the same larval and adult diets, in the same laboratory, possessed similar microbiota [37]. Indeed, similar bacteria were also identified from *B. neohumeralis* laboratory-adapted colonies, which were established 3 years apart but reared within the same facility [37]. Interestingly, the gut microbiome profile of *B. neohumeralis* differed between populations reared in different laboratories, suggesting an environmental influence on the bacteria associated with artificially reared-adult fruit flies. Identifying the factors driving changes in tephritid gut microbiota, such as age, diet, environment and genetics, is important to identify ways to minimise, or even avoid, unwanted microbial changes, and optimise the gut ecology of mass-reared tephritids.

When domesticated flies are stressed due to nutrition, overcrowding, increased waste products, exposure to larger densities of particular bacteria and genetic changes, this could influence fly susceptibility to pathogens. For example, *Serratia marcescens* is pathogenic to *Rhagoletis pomonella* [79] and to *Drosophila melanogaster* [80, 81]. Lloyd et al. [69] found that Enterobacteriaceae, such as *Klebsiella*, *Erwinia* and *Enterobacter*, were frequently cultured from field collected *B. tryoni*, while *S. marcescens* and *Serratia liquefaciens* were dominant in laboratory flies, which may have been introduced by *D. melanogaster* flies frequently found around laboratory-reared tephritid colonies. Mortality of *Bactrocera jarvisi* larvae feeding on a carrot diet at neutral pH spiked with *S. liquefaciens*, suggests that this bacterium can be pathogenic [26]. In a recent study, *Serratia* spp. were shown to dominate (> 90%) a laboratory-adapted *B. cacuminata* colony, relative to a wild population, which was dominated (> 90%) by *Enterobacter* spp. [37]. In the same laboratory, > 60% of the bacteria within laboratory-adapted *B. jarvisi* comprised of *Serratia* spp. [37]. These organisms were also detected (but were not dominant) in laboratory-adapted *B. tryoni* sourced from different populations, but formed only a very minor constituent of field-caught *B. tryoni* [37]. Further work is required to determine whether the genus *Serratia*, members of this genus, or relative amounts of *Serratia* are pathogenic to tephritids fed artificial diets, or whether it is the loss of important endosymbionts as a result of the presence of *Serratia* that negatively impacts the host. This also highlights the need to better understand the interplay between tephritid microbes.

Good sanitary procedures within a mass-rearing facility are essential. Many aspects of the mass-rearing environment, such as communal feeding, encourage the spread of pathogens, which could be bacterial, viral, fungal or protozoan. Within a laboratory setting, bacteria can spread from adjacent cages within days, to several meters within a few weeks [72]. They could also be spread through equipment and staff maintaining fly colonies. However, it is not just pathogenic bacteria that can be harmful to fruit fly rearing efforts, but also the presence of unwanted microbes in the diet that could deplete nutrients, increase fermentation within the diet, or produce metabolic wastes that are harmful or repulsive to fruit flies [78]. Such effects add to the stress of rearing, which in turn may increase susceptibility to pathogens [82]. Furthermore, some microbial contaminants could be harmful to production facility staff, farmers and in the end consumers when the flies are released, plus the plants the flies come into contact with as fruit flies are capable of spreading bacteria in the field [83]. However, maintenance of beneficial gut bacteria of flies through dietary and environmental manipulation, or the use of probiotic diet supplements to encourage beneficial microbes, and restrict pathogens or contaminants in the fruit fly diet are areas of research that demonstrate great potential.

The general hypothesis is that microbial diversity contributes to healthier flies and that observed taxonomic differences in artificially reared flies result in less resilience and increased sensitivity to environmental changes due to decreased bacterial diversity and perhaps decreased functional diversity. Little is known about the relationship between the structure of fruit fly bacterial communities and functional diversity and the impact of taxonomic differences at the functional level. Analytical approaches such as metagenomics, metatranscriptomics, metabolomics and metaproteomics will facilitate significant progress in this area as they will permit better characterization of microbial communities, their function and contribution to host development, fitness and performance.

### Effect of irradiation

Fruit flies to be released in SIT operations are typically sterilized as pupae using gamma irradiation [84]. Lauzon and Potter’s [85] comparison of irradiated versus non-irradiated *C. capitata* and *A. ludens* midguts using electron microscopy showed that irradiation has an effect on both the gut microbiota and the development of the midgut epithelium. Transmission electron microscope images revealed that bacteria in two-day old flies irradiated as pupae appeared to be irregular in shape and lacked fimbriae, while the bacteria in non-irradiated two-day old flies attached to the peritrophic membrane using fimbriae. Irradiation also had an effect on peritrophic membrane development, which appeared to be irregular and gel-like in two-day old irradiated flies, while it was well developed in two-day old non-irradiated flies [85]. It is not yet known whether this tissue and bacterial cell damage was long-lasting and warrants further investigation. Damage to the gut epithelium may not only have an effect on the gut bacterial population, but may consequently also affect nutrient absorption. Although the gut bacterial community structure was different in newly eclosed irradiated compared to non-irradiated mass-reared Vienna 8 *C. capitata*, after 5 days the gut community resembled the gut community of non-irradiated flies at eclosion [56]. This difference at emergence could reflect a change in nutrient availability and uptake to an irradiated fly during the first few days after eclosion. The study by Lauzon et al. [21] demonstrated that irradiation does not appear to disrupt the vertical transmission of *E. agglomerans* and *K. pneumoniae*, originally coated onto fruit fly eggs, to adult *C. capitata* guts and it is currently unclear how irradiation actually induces a shift in gut bacterial community. However, we can hypothesize that damage caused by irradiation to the gut epithelium and bacteria, in addition to the associated stress, can influence the gut bacterial community. In *Drosophila* gut microbes stimulate intestinal stem cell activity, which can renew the gut epithelium [86]. As gut bacteria influence epithelial metabolism and cell proliferation through the production of short chain fatty acids, such as acetate, butyrate and propionate [87, 88], greater knowledge of this in tephritids may identify ways to improve the ability of mass-reared flies to recover from irradiation.

### Potential of probiotics to improve tephritid performance

**Fig. 1:**
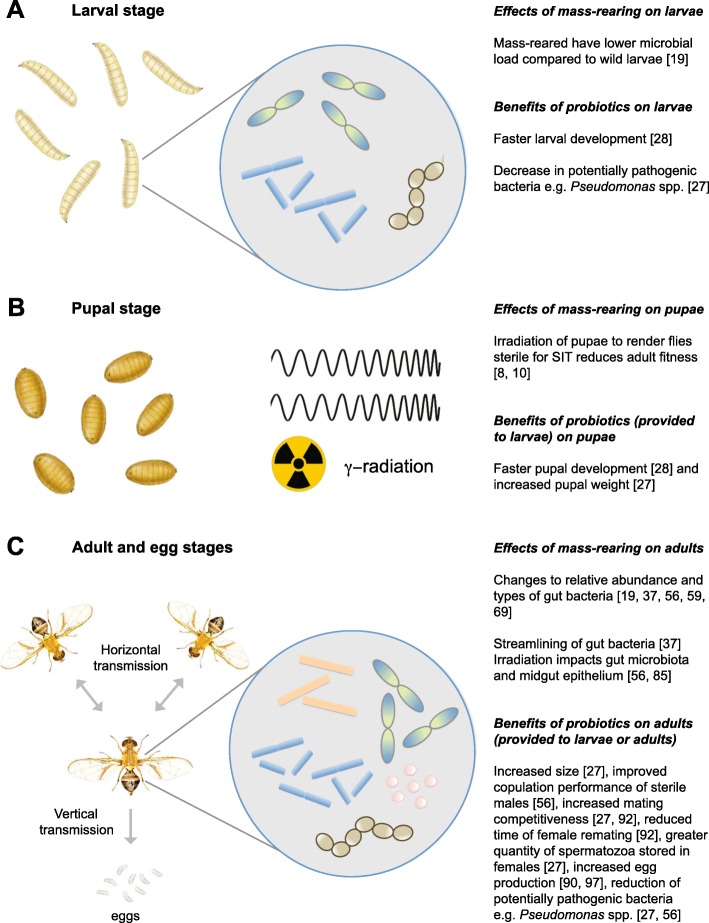
Tephritid life stages, the effects of mass-rearing on the gut microbiome, and the benefits of probiotic applications to the diet. **a** Larval stage with representation of the bacterial gut microbiome; **b** pupal stage, which is treated with gamma irradiation for the sterile insect technique (SIT); **c** adult and egg stages with representation of the adult gut microbiome. Larval and pupal illustrations adapted from Hely et al. [97] and adult illustration adapted from the Australian Insect Names Website by the Commonwealth Scientific and Industrial Research Organisation and Department of Agriculture and Fisheries [98]

Probiotics, by definition, refers to products that contain a sufficient number of live microorganisms that confer a discernible health benefit on the host [89]. Drew et al. [90] conducted one of the first studies demonstrating that bacteria are a food source and provide nutrients to fruit flies, which, in turn, can positively influence egg production and longevity. Since then, over ten studies [26–28, 56, 72, 91–96] have investigated the effect of bacterial supplements, more recently referred to as probiotics, added to tephritid diets, on the host, with mixed results. Substantial changes are not always observed (discussed below); however, the majority of the changes recorded positive outcomes for the host (Fig. 1). Measurements of tephritid fruit fly fitness and performance following administration of probiotics mostly focus on immediate benefits. Therefore, it is possible that other impacts, such as changes in the expression of host immune response genes, and genes involved in signalling and/or metabolism, have been overlooked. Negative impacts have been observed in probiotic fed adult *B. oleae*, where a reduction in longevity has been observed; however, whether this appears to be influenced by the diet the adult flies are feeding on (i.e. sugar versus sugar and protein diet) or the bacteria remains unclear [99]. The bacterial species fed to the adult fly can also influence longevity [90]. Thus, benefits provided by probiotics are not always consistent between studies, most likely due to the complexity of tephritid-bacteria interactions. Further, other factors are likely to influence results including variations in experimental design, probiotic supplements tested and their delivery (dose, mode), experimental conditions, traits measured on varying life stages, irradiated or non-irradiated flies, pre-existing microbiota in experimental flies, diet (nutritional value, antimicrobials, agar versus granular), rearing environment, age and genetic diversity of experimental colonies. As the wild tephritid gut microbiome is often comprised of diverse microbiota, it is feasible that the addition of more than one probiotic candidate, i.e. bacterial blends/consortiums, to domesticated tephritids may provide increased or even additional benefits. Therefore, any probiotic study needs to be well replicated, or a sufficient number of samples included due to the complexity of such studies. In addition, any trade-offs (if observed) need to be assessed, for example, against improved mating performance, as to their importance in SIT effectiveness.

The addition of symbiotic bacteria to the larval and adult fruit fly diets changes the structure of fruit fly gut bacterial communities (Fig. 1). Indeed, adding a probiotic supplement cocktail containing *Klebsiella pneumoniae*, *Enterobacter* sp. and *Citrobacter freundii* to the *C. capitata* larval diet simultaneously increased the number of Enterobacteriaceae in the larval and adult gut and reduced the number of *Pseudomonas* spp. present at both the larval and adult stages [27]. Similarly, feeding *Klebsiella oxytoca* to adult Vienna 8 strain *C. capitata* increased the abundance of *K. oxytoca* in the gut, and reduced the number of *Pseudomonas*, *Morganella* and *Providencia* spp. [56]. It is hypothesized that the gut Enterobacteriaceae community of *C. capitata* can control the density of bacteria that are harmful in high abundance, such as *Pseudomonas aeruginosa* [54].

The majority of tephritid probiotic studies have involved the addition of bacteria to the adult diet, and while, the observed impacts on the host have been variable, the positive impacts are encouraging for their potential application in SIT programs (Fig. 1). Sterile male *C. capitata* fed a sugar diet enriched with *K. oxytoca* compared to flies fed a sugar only diet, showed increased mating competitiveness in both laboratory and field cages, reduced female remating (presumably in a laboratory setting), and increased survival under starvation in the laboratory [92]. Similarly, a mating advantage was conferred in laboratory studies of *C. capitata* fed *Enterobacter agglomerans* and *K. pneumoniae* in a yeast-enhanced agar compared to non-bacterial inoculated yeast-enhanced agar, but no significant effect was observed with a sugar-yeast or sugar-reduced yeast granular diet [94]. Conversely, mating competitiveness studies in field cages only found significantly more matings (with wild/F1-F15 laboratory-reared flies) than the control when the flies were fed yeast-reduced sugar granulate diet [94]. While mating was not assessed, Meats et al. [93] detected no evidence of either, *K. oxytoca* or *K. pneumoniae* added to the adult *B. tryoni* diet (paste of sugar and autolysed yeast) impacting on egg production regardless of whether the fly generation was F0-F20; however, as expected (presumably due to laboratory adaptation) regardless of bacterial supplementation, egg production increased as the fly generation increased. The addition of *Pseudomonas putida* to the sugar diet of *B. oleae* increased female fecundity compared to females fed a sugar only diet [99]. However, *P. putida* added to a complete diet (comprising of sugar and hydrolysed brewer’s yeast) had no significant effect on fecundity compared to the same diet without added *P. putida* [99]. These studies indicate that bacteria contribute to fly nutrition, although not exclusively (see next paragraph). It is possible that when the flies are provided with a nutritionally balanced diet, i.e. the amount of yeast, providing fatty acids, amino acids and vitamins, is adequate, the effect of a probiotic supplement is minimal, but this would be dependent on the influence of nutrition on the trait being measured. Thus, the role of the gut microbiome may have largely been underestimated in nutritional studies, and it is possible that through adding bacterial supplements to the fruit fly diet, the amount of yeast required could be reduced. Further, other components of the gut microbiome, such as yeasts, which can also contribute to the host nutrition, have largely been overlooked until recently [100].

Several studies have investigated the impact of feeding autoclaved bacteria, which by definition are not classed as a probiotic, to tephritids [28, 56, 92]. Autoclaved *Enterobacter* sp. added to the *C. capitata* larval diet significantly reduced egg to adult developmental time [28]. This study suggests that bacterial mass and/or bacterial substrates can have a positive nutritional effect on immature *C. capitata.* However, studies comparing the use of autoclaved bacteria to live bacteria show that the contribution of live bacteria to the host is greater than just the nutritional value of dead bacteria themselves and what they produce in culture. The addition of autoclaved *K. oxytoca* to the *C. capitata* adult pre-release sugar/sucrose only diet did not improve mating performance [92] or mating latency [56], in contrast to a diet supplemented with live *K. oxytoca*. The nutritional benefits observed when using an autoclaved, or live culture may be due to metabolites produced by the bacteria; it is not known what metabolites are being produced by tephritid gut bacteria and what effect they have on the gut microbiome and the host. In *Drosophila*, the metabolite acetate, a product of pyrroloquinoline quinone–dependent alcohol dehydrogenase (PQQ-ADH) by the commensal gut bacterium, *Acetobacter pomorum*, modulates insulin/insulin-like growth factor signalling, which is important for normal larval development [101]. Gut microbiota and their metabolites will be an exciting area of research to follow in the future, particularly with the development of tools such as metabolomics.

Although only a few studies have investigated the effects of adding probiotic supplements to the larval diet, the results have revealed a number of benefits. Addition of *Enterobacter* spp., *K. pneumoniae* and *C. freundii* to the wheat bran larval diet increased pupal weight of *C. capitata* Vienna 8 genetic sexing strain (GSS), fly size, spermatozoa storage in females, and also improved aspects of mating competitiveness of sterile flies in laboratory settings [27]. The addition of an *Enterobacter* sp. to the larval carrot-based diet improved egg-pupal and egg-adult recovery of *C. capitata* Vienna 8 GSS, and reduced the duration of the egg-pupal, pupal and egg-adult stages [28]. Reduced developmental time is a considerable advantage in mass-rearing facilities leading to cost savings and increased production. The benefits observed at the larval stage could have flow-on effects to the pupae and to adult morphology, fitness and performance. Thus, there is a need to increase our understanding of the influence of each life stage on successive stages and generations, particularly considering the vertical transmission of microbiota.

The presence of beneficial microbes in the larval diet may allow a reduction in the added amount of antimicrobials. Some yeasts possess antagonistic properties against undesirable bacteria [102]. Four studies have cultured yeasts from field collected tephritid fruit fly larvae (*B. tryoni* and *Anastrepha mucronota*) indicating that they consume yeasts while feeding within fruit [100, 103–105]. Thus, the incorporation of live yeasts, rather than pasteurized yeasts, for example, into the larval diet may be a way to reduce the amount of antimicrobials in the diet and warrants further testing. The interaction between bacteria and yeasts in the gut is an unexplored area in tephritid fruit fly research.

The development of a tephritid gnotobiotic model system that allows the addition and manipulation of flies, which have either developed under axenic conditions, or for which all present microbiota are known, would enable the better examination and verification of host-microbe relationships. Surface sterilization of eggs would remove the transmission of gut microbiota transferred with the egg during oviposition, and the larvae that emerge can then be used in an axenic system. This would help avoid non-microbial effects that could derive from the use of antibiotics, such as effects on mitochondrial respiration [106].

## Conclusion

While significant progress has been made towards the taxonomic characterisation and profiling of gut microbial populations in tephritids, there are still considerable gaps in our knowledge of tephritid-bacteria interactions. Improvements in NGS technologies and bioinformatics, in combination with decreased costs, will improve our knowledge of gut microbial diversity and potentially identify further key bacterial and other microbial symbionts. However, the largest unknown factors remain with the functional roles of the microbial symbionts. Use of gnotobiotic models, genome sequencing, metagenomics, metatranscriptomics, metabolomics and metaproteomics will help in defining precise roles of gut microbes. To maintain tephritid-microbial symbiont interactions during the mass-rearing process, we need to understand how such interactions evolve and how both irradiation [107] and the domestication process [108], including diet, disrupts the relationship and associated bacterial functions. This will inform the development of ways to encourage, maintain or introduce symbiotic microbes in the rearing process to produce better performing, and cost-effective flies for SIT programs. Microbial symbionts, whether through the administration of larval and/or adult probiotics, or the maintenance of a healthy gut microbiome through dietary and environmental manipulation, may well be the next major improvement to fruit fly mass-rearing.

## Data Availability

Not applicable.

## Abbreviations

AW-IPM: Area wide - integrated pest management
GSS: Genetic sexing strain
NGS: Next-generation sequencing
OTU: Operational taxonomic unit
PQQ-ADH: Pyrroloquinoline quinone–dependent alcohol dehydrogenase
SIT: Sterile insect technique
